# Which therapy works best for maternal depressive symptoms? A network meta-analysis of psychotherapeutic interventions

**DOI:** 10.1007/s00737-025-01658-y

**Published:** 2026-01-08

**Authors:** Hakan Öztürk, Rüveyda Yüksel, Ayça Balmumcu

**Affiliations:** 1 Faculty of Medicine, Aydın Adnan Menderes University, Aydin, Turkey; 2 Faculty of Nursing, Aydın Adnan Menderes University, Aydin, Turkey; 3 Söke Vocational School of Health Services, Aydın Adnan Menderes University, Aydin, Turkey

**Keywords:** Network meta-analysis, Maternal depression, Psychotherapeutic intervention, Comparative efficacy

## Abstract

**Purpose:**

Maternal depression is a significant public health concern that can adversely affect both mothers and their children. Although various psychotherapeutic interventions have been proposed, their relative comparative efficacy remains unclear. This network meta-analysis (NMA) aimed to evaluate and compare the efficacy of different psychotherapeutic interventions in reducing maternal depressive symptoms.

**Methods:**

A systematic search was conducted in the Web of Science Core Collection (Science Citation Index Expanded and Social Sciences Citation Index) to identify randomized controlled trials (RCTs) published between 1 February 2021 and 1 February 2025. Eligible studies included mothers aged ≥ 18 years who were assessed for maternal depressive symptoms using the Edinburgh Postnatal Depression Scale (EPDS) and received any form of psychotherapy. Data were extracted using a predefined format (PROSPERO ID: CRD420251010916). Random-effects models were used to perform the NMA in R, reporting mean differences (MD) with 95% confidence intervals (CIs) and P-scores.

**Results:**

A total of 8 RCTs involving 2,919 participants were included. Cognitive behavioral therapy (CBT) was the only intervention that showed a statistically significant reduction in depressive symptoms compared to treatment as usual (TAU) (MD = -3.22, 95%CI: -5.91 to -0.54; *p* = 0.019; P-score = 0.92). Other interventions showed trends toward improvement, but these were not statistically significant (*p* > 0.05).

**Conclusion:**

CBT emerged as the most efficacious psychotherapeutic approach in both direct and indirect comparisons, supported by statistical evidence from the NMA.

## Highlights

This study provides comparative efficacy evidence to inform treatment guidelines for maternal depression.

CBT was the most efficacious intervention among the psychotherapeutic approaches examined.

Findings support prioritizing CBT in clinical care and future maternal mental health guidelines.

## Introduction

Maternal depression is defined as a major depressive disorder that occurs during pregnancy or after childbirth, and it has negative effects on mother-child health and family functioning (Byatt et al. 2016). The prevalence of maternal depression ranges from 10% to 14% during the prenatal period 8 during the prenatal period and 13–17% during the postpartum period, with prevalence rates reaching up to 40% among women in low-income and minority groups (Dennis et al. 2017; Yildiz et al. 2017; Alhusen et al. 2021). This prevalence indicates that maternal depression is not only an important issue at the individual level but also a significant public health concern.

Various adverse outcomes such as breastfeeding problems, anxiety, and impaired parenting behaviors have been observed in women experiencing maternal depression (Hunter et al. 2024). Additionally, maternal depression is associated with developmental risks in children, such as poor social interaction, emotional problems, and insecure attachment (Priel et al. 2020; Hunter et al. 2024). Furthermore, maternal depression is associated with unhealthy behaviors such as irregular prenatal care, poor nutrition, smoking, alcohol, and substance use, as well as intrauterine growth restriction and the risk of partner depression (Underwood et al., 2017; Yasuma et al. 2019). In the postpartum period, child neglect, abuse, and infant mortality can pose serious threats to child health (Takehara et al. 2017; Brockington 2017). These multifaceted effects highlight that maternal depression is a critical health issue that must be prevented and effectively treated at the levels of women’s health, mental health, and public health (Don and Mickelson 2012; Paulson and Bazemore 2010).

Despite maternal depression being recognized as a widespread problem worldwide and posing a serious burden on maternal and child health, a significant proportion of women do not have access to effective treatment. Barriers to treatment include difficulties in accessing health services, economic constraints, individual beliefs, social stigma, and cultural factors (Place et al. 2024). In addition, many women are reluctant to use medication during pregnancy and breastfeeding, which limits the use of pharmacological treatment options (Webb et al. 2024). In this context, it is important to examine the effectiveness of psychotherapeutic approaches in the treatment of maternal depression. Psychotherapeutic interventions offer a safe alternative for women who do not wish to use medication and also stand out as a more widely accepted intervention method at both the individual and societal levels (Singla et al. 2025).

Cognitive behavioral therapy (CBT), interpersonal therapy (IPT), supportive therapy, psychodynamic therapy, mindfulness-based approaches, and group therapies are among the psychotherapeutic approaches used in the treatment of maternal depression. Each of these therapeutic approaches can be effective in alleviating the symptoms of maternal depression; however, findings in the literature regarding which approach is superior vary (Cuijpers et al. 2023). While traditional meta-analyses are limited to directly comparing only two treatment methods, network meta-analysis (NMA), a more statistically advanced method, enables direct and indirect comparisons of multiple interventions simultaneously, providing more comprehensive results. This method provides analyses that compare the effectiveness of therapeutic interventions using statistical models, thereby grounding clinical decision-making processes in more robust foundations. In the treatment of complex and multidimensional mental health issues such as maternal depression, the NMA method provides valuable insights not only from a clinical perspective but also from an epidemiological and statistical standpoint (Salanti 2012). This analytical approach provides evidence-based decision support to healthcare professionals by considering not only direct differences between treatments but also indirect effects (Cipriani et al. 2013). Clinical guidelines recommend priority screening and treatment options for maternal depression. However, these guidelines do not provide specific recommendations on which treatments are most effective. Many psychotherapeutic interventions previously evaluated have been found to be effective in reducing depression symptoms when compared to standard care or placebo (O’Connor et al., 2019). However, no statistically based study has been found that directly and indirectly compares these interventions with each other. In this context, this study, conducted using NMA, aims to systematically evaluate the relative effectiveness of different interventions. This statistical approach will enable healthcare professionals to make more objective, data-driven, and informed decisions regarding the intervention to be used in the treatment of maternal depression; it will also provide strong scientific evidence regarding the most appropriate approach. Accordingly, the aim of this study is to evaluate the comparative efficacy of different psychotherapeutic interventions used to reduce maternal depressive symptoms using the NMA method.

## Materials and methods

This study was conducted in accordance with the Preferred Reporting Items for Systematic Reviews and Meta-Analyses (PRISMA) guidelines recommended for reporting systematic reviews and network meta-analyses (Phillips and Barker 2021). The study protocol was registered in the International Prospective Register of Systematic Reviews (PROSPERO) database under registration number CRD420251010916.

### Search strategy

The literature review was conducted in the Web of Science (WoS) Core Collection, including the Science Citation Index Expanded (SCI-EXPANDED) and Social Sciences Citation Index (SSCI). This database was selected because it provides comprehensive coverage of peer-reviewed and indexed international journals across medical and psychological disciplines, minimizing duplication across other databases such as PubMed, PsycINFO and Embase. Searches were limited to studies published between 1 February 2021 and 1 February 2025, to capture the most recent evidence on psychotherapeutic interventions for maternal depressive symptoms. Searches were conducted between March 13 and April 13 2025, and only studies written in English were included. The following keywords were used in the search strategy:

- (“maternal depression” OR “depression”) AND (“pregnan*” OR “postpartum”) AND (“therapy”).

The full query used in the Web of Science database can be accessed at the following link:

https://www.webofscience.com/wos/woscc/summary/4c85d6e1-d892-45e4-90ea-a69039fff54e-014d183869/relevance/1.

The reference lists of the studies included to broaden the scope of the relevant literature were also searched.

## Eligibility and exclusion criteria

Inclusion and exclusion criteria were determined based on the PICOS model (Eriksen and Frandsen 2018).

Eligibility criteria:

1. Population: Women aged 18 years and older experiencing maternal depressive symptoms.
2. Intervention: Any psychotherapeutic intervention method (no restrictions on therapy type, frequency, or duration).
3. Comparison: Usual care (treatment as usual-TAU) or psychotherapeutic intervention methods.

In this study, Usual Care (Treatment as Usual, TAU) refers to routine perinatal or postpartum care provided by healthcare professionals, which may include general health education, brief counseling, and regular follow-up, but does not involve structured psychotherapy or manualized psychological interventions. This definition was applied consistently across the included studies to ensure comparability of intervention effects.

4. Outcome: Reduction in maternal depressive symptoms, primarily assessed using the Edinburgh Postnatal Depression Scale (EPDS). The EPDS provides a standardized quantitative measure of symptom severity and was selected to ensure methodological consistency and comparability across trials. It is one of the most validated and widely used instruments for assessing depressive symptoms during the perinatal and postpartum periods (Gibson et al. 2009; Levis et al. 2020). Although not a diagnostic tool, the EPDS enables standardized quantification of symptom severity, facilitating quantitative synthesis across studies.
5. Study Type: Randomized controlled trials (RCTs).

Exclusion criteria:

1. Studies that did not measure maternal depressive symptoms as a primary or secondary outcome, or lacked quantitative EPDS data, were excluded.
2. Non-randomized or quasi-experimental studies (e.g., reviews, conference abstracts, protocols, case–control and cohort studies, unpublished theses, and non–peer-reviewed works) were excluded.
3. Articles excluded during the title/abstract screening phase did not involve psychotherapeutic interventions for maternal depressive symptoms, whereas those removed after full-text assessment lacked sufficient outcome data or did not meet methodological inclusion criteria. In total, 226 studies were excluded during title/abstract screening and 13 after full-text review.

## Data management, selection, and screening

The screening and data extraction processes were carried out by two independent researchers (AB & RY). These processes were conducted systematically to ensure consistency and transparency throughout the review. Disagreements were resolved through discussion, and the final decision was reached by consensus. First, titles and abstracts were screened, and full texts were evaluated according to eligibility criteria. In case of any inconsistency, a third independent researcher (HÖ) performed the evaluation.

**Fig. 1:**
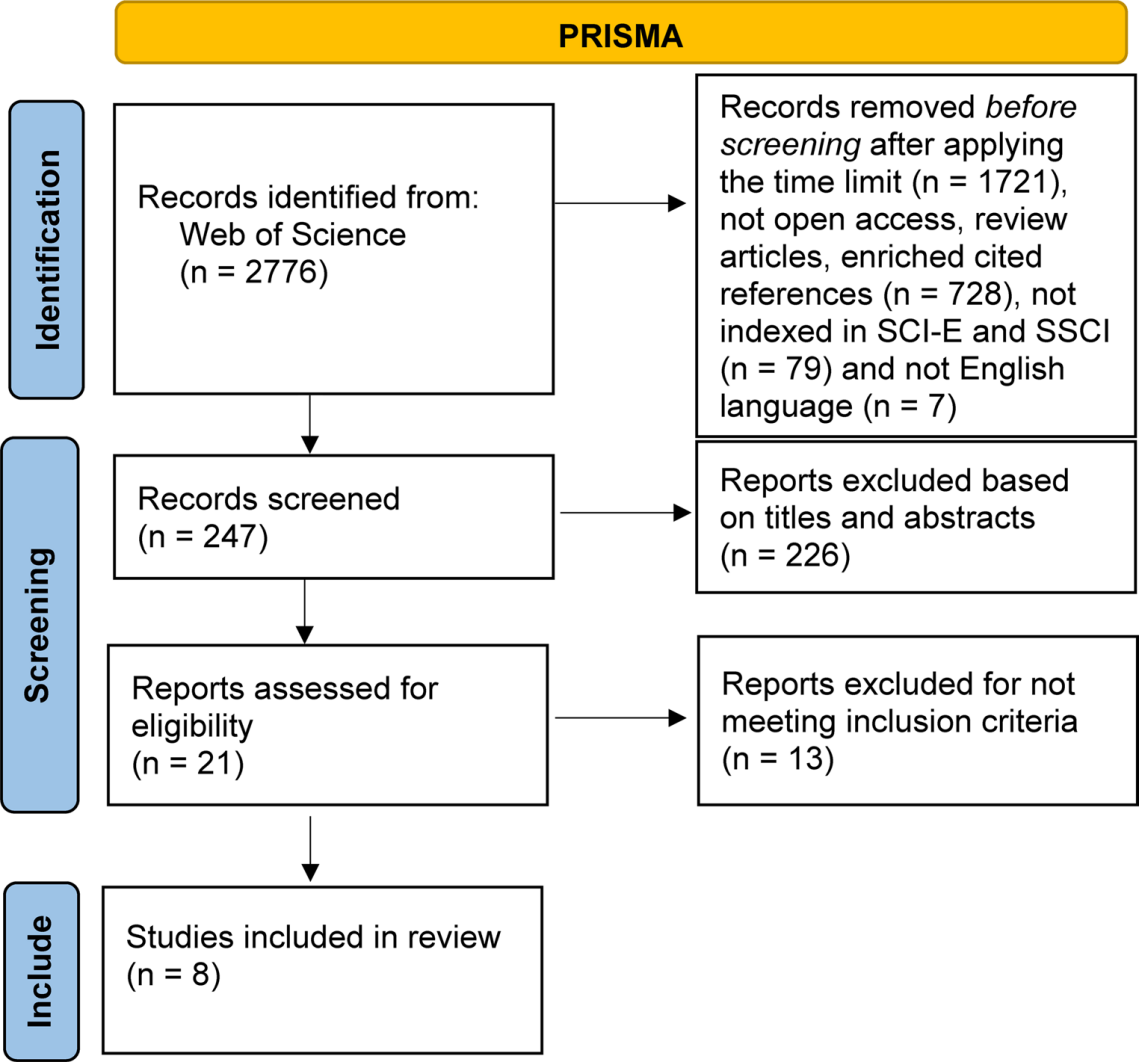
PRISMA flow chart

Initially, 2,776 studies were identified through database searches. Following the application of a publication date restriction, 1,721 studies were excluded, resulting in 1,055 articles eligible for further review. Subsequently, articles that were not open-access, review articles, or enriched cited references (*n* = 728), those not indexed in SCI-E and SSCI (*n* = 79), and those not published in English (*n* = 7) were excluded, leaving 274 articles for further evaluation. A total of 121 non–open access articles were excluded because their full texts could not be retrieved through institutional or public access channels. This exclusion criterion was applied solely due to data unavailability, not study quality. We acknowledge this as a limitation of the review, as excluding non–open access studies may have led to the omission of potentially relevant evidence. Upon screening the titles and abstracts, an additional 226 articles were excluded. Of the remaining 21 potentially eligible studies, a total of eight articles met the inclusion criteria and were included in the NMA. Screening and study selection are shown in Fig. 1 using the PRISMA flow diagram.

A data extraction tool developed by researchers was used to obtain the data for the study. This data extraction tool was used to obtain data on authors and publication years, interventions applied, study design, sample size, data collection tools, and statistical results (Table 1).

**Table 1:** Characteristics of included studies

References (country)	Sample (I/C)	Intervention (I/C)	Details of interventions	Measurement Tools	Statistical Results EPDS Score (Mean ± SD)
Leng et al. (2023) China	75 (38/37)	MDMBI/TAU	8 weekly sessions	EPDS, PSS, FFMQ, SCS, BMSWB (PA)	I: 5.70 ± 3.82, C: 8.90 ± 3.77
Merza et al. (2024) Canada	137 (71/66)	CBT/TAU	9 weekly 2-hour sessions	EPDS, GAD-7	I: 10.20 ± 4.32, C:13.96 ± 4.90
Park et al. (2025) Republic of Korea	94 (46/48)	MDMBI/TAU	4 weekly 20-min sessions, repeated twice daily	DASS, EPDS, MHC-SF, MFAS, CAMS-R	I: 6.20 ± 4.60, C:8.30 ± 6.0
Amani et al. (2023) Canada	48 (25/23)	CBT/TAU	9 weekly 2-hour sessions	EPDS	I: 10.60 ± 4.20 C: 16.80 ± 5.20
Carona et al. (2023) Portugal	1053 (542/511)	WBI/TAU	5–8 weeks (self-paced, 1 module/week recommended)	EPDS, HADS-A, DERS	I: 8.75 ± 4.53, C: 10.78 ± 5.06
Gaden et al. (2022) Argentina, Colombia, Israel, Norway, and Poland	213 (105/108)	MT/TAU	3 weekly sessions (~ 30 min each)	GAD-7, EPDS, PBQ	I: 6.02 ± 4.45, C: 6.20 ± 5.21
Sun et al. (2021) U.S.A., China	168 (84/84)	MDMBI/WeChat-BHC	8 weekly sessions (~ 20 min each)	EPDS	I: 7.99 ± 3.87, C: 8.55 ± 4.59
Donegan et al. (2022) Canada	75 (40/35)	CBT/TAU	6 weekly sessions	EPDS, STICSA	I: 6.86 ± 3.73, C: 7.13 ± 3.47

MDMBI: Mobile-Delivered Mindfulness-Based Intervention, TAU: Treatment As Usual, CBT: Cognitive Behavioral Therapy, WBI: Web-Based Intervention MT: Music Therapy, WeChat-BHC: WeChat-based Health Consultation, C): I: Intervention group, C: Control group, SD: Standard Deviation, EPDS: Edinburgh Postnatal Depression Scale, PSS: Perceived Stress Scale, FFMQ: Five Facet Mindfulness Questionnaire, SCS-SF: Self-Compassion Scale – Short Form, BMSWB (PA): Body-Mind-Spirit Well-Being Inventory–Positive Affect subscale, GAD-7: Generalized Anxiety Disorder Questionnaire – 7 items, DASS: Depression, Anxiety and Stress Scale, MHC-SF: Mental Health Continuum–Short Form, MFAS: Maternal-Fetal Attachment Scale, CAMS-R: Cognitive and Affective Mindfulness Scale–Revised Version, HADS-A: Hospital Anxiety and Depression Scale–Anxiety Subscale, DERS: Difficulties in Emotion Regulation Scale, PBQ: Parenting Bonding Questionnaire, STICSA: State-Trait Inventory for Cognitive and Somatic Anxiety

Mean ± SD values represent post-intervention EPDS scores, unless otherwise specified. In studies reporting change scores, the mean difference from baseline to post-intervention was extracted

### Evaluation of the methodological quality of studies

The methodological quality of the studies included in this systematic review was assessed using checklists published by the Joanna Briggs Institute. Accordingly, the quality assessment of randomized controlled studies was performed using a 13-item checklist (Barker et al. 2023; Tufanaru et al. 2017). Each item on these lists is evaluated as “yes, no, uncertain, or not applicable.” The methodological quality level of the studies included in the research was considered “poor” if less than 50% of the items were evaluated as “yes,” “moderate” if 51–80% of the items were evaluated as “yes,” and ‘good’ if more than 80% of the items were evaluated as “yes.” The results of this methodological quality assessment for the eight included randomized controlled trials are summarized in Table 2.

**Table 2:** Quality assessment of the included studies

	Item 1	Item 2	Item 3	Item 4	Item 5	Item 6	Item 7	Item 8	Item 9	Item 10	Item 11	Item 12	Item 13	Total	Quality
Leng et al. (2023)	1	1	1	0	0	0	1	1	1	1	1	1	1	10	Moderate
Merza et al. (2024)	1	1	1	0	0	1	1	1	1	1	1	1	1	11	High
Park et al. (2025)	1	1	1	0	0	1	1	1	1	1	1	1	1	11	High
Amani et al. (2023)	1	0	1	0	0	0	1	1	1	1	1	1	1	9	Moderate
Carona et al. (2023)	1	1	1	0	0	0	1	1	1	1	1	1	1	10	Moderate
Gaden et al. (2022)	1	1	1	0	0	0	1	1	1	1	1	1	1	10	Moderate
Sun et al. (2021)	1	1	1	0	1	1	1	1	1	1	1	1	1	11	High
Donegan et al. (2022)	1	1	1	0	0	1	1	1	1	1	1	1	1	11	High

### Statistical analysis

Statistical analyses were performed using the “netmeta” library in R software (version 4.4.2). The NMA was performed using a random effects model to conduct direct and indirect comparisons between psychotherapeutic interventions. Estimated mean differences between different intervention methods and their confidence intervals were visualized using a forest plot. The relative treatment efficacy ranking of the interventions was determined using the area under the cumulative ranking curve (SUCRA) values. Funnel plot analysis was performed to assess publication bias and small study effects. Heterogeneity among studies was assessed and interpreted using the Q statistic, I², and τ² values. The R script used for the NMA (including data processing and model specifications) is available from the corresponding author upon reasonable request to ensure transparency and reproducibility.

## Results

### Study characteristics

Table 1 shows the characteristics of the eight RCTs included in the study. These studies were published between 2021 and 2025. A total of 1,863 participants were evaluated in the studies, and six different intervention methods were used in the analysis. The psychotherapeutic intervention methods used were Cognitive Behavioral Therapy (CBT), Mindfulness Therapy (MDMBI), Music Therapy (MT), Standard Treatment (TAU), Web-Based Intervention (WBI), and WeChat health counseling (WeChat-BHC).

## Network structure

**Fig. 2:**
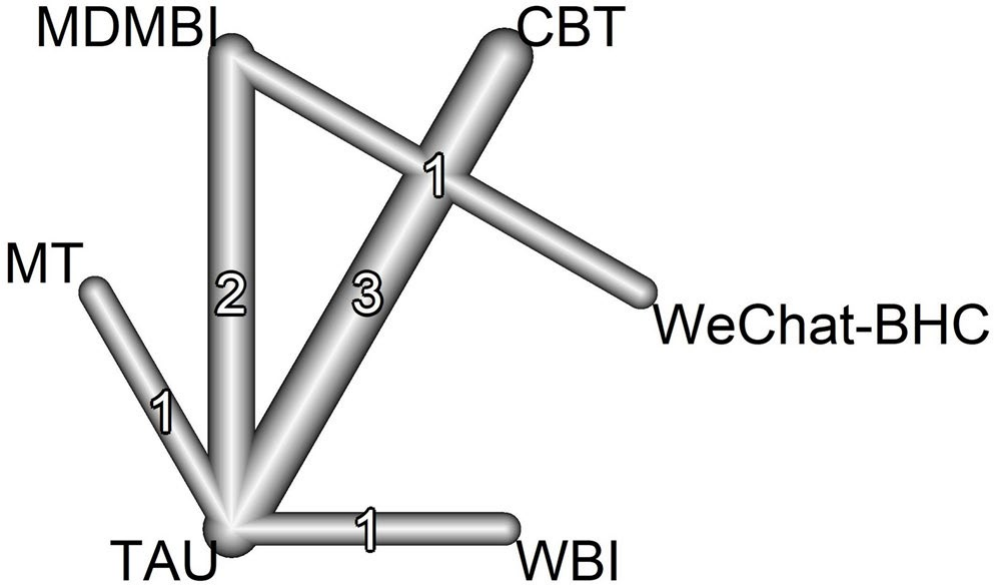
Network diagram

In this NMA comparing the efficacy of psychotherapeutic interventions in the treatment of maternal depression, a total of eight pairwise comparisons were made, and these comparisons were in five different study designs. Fig. 2 shows the network diagram of the intervention groups included in the study. Fig. 2 shows the connections between the types of therapy included in the studies evaluated using the EPDS. As seen in the diagram, standard treatment (TAU) is the most frequently compared treatment method.

### Heterogeneity assessment

According to the heterogeneity assessment performed in the NMA, significant heterogeneity was detected among the studies (Cochran Q = 18.29; *p* < 0.001; τ² = 4.650; I² = 83.6%). This result showed that there were significant differences in maternal depression outcomes among the studies included in the analysis. The literature indicates that the random effects model should be used when heterogeneity is detected in NMA studies (Chaimani et al., 2017; Salanti 2012; Higgins et al. 2009). Therefore, the effects of interventions on maternal depressive symptoms were evaluated using the random effects model in the NMA.

### Pairwise comparisons of interventions

Table 3 presents comparative results regarding the effects of other interventions on maternal depressive symptoms, with TAU as the reference. Upon examining Table 2, CBT resulted in a significant decrease in depressive symptoms compared to TAU (MD=−3.22, *p* = 0.019). Although other interventions showed a tendency toward a decrease in depressive symptoms, they did not result in a statistically significant decrease compared to TAU (*p* > 0.05).

**Table 3:** Effect of psychotherapeutic interventions on depressive symptoms

Treatment	MD	95% CI	*p*
CBT	−3.22	[−5.91, −0.53]	**0.019**
MDMBI	−2.67	[−5.96, 0.62]	0.111
MT	−0.18	[−4.60, 4.24]	0.936
WBI	−2.03	[−6.30, 2.24]	0.351
WeChat-BHC	−2.11	[−7.62, 3.40]	0.452

*MD*: mean difference, *CI*: confidence interval

**Fig. 3:**
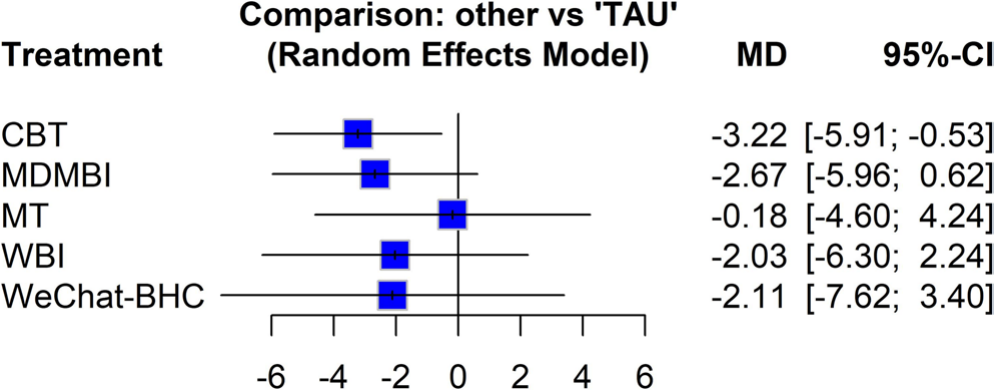
Forest plot

The forest plot in Fig. 3 shows the mean differences in EPDS scores between TAU (reference) and other interventions, along with their 95% confidence intervals. Negative values indicate a decrease in EPDS scores and, consequently, a reduction in depression symptoms. According to the forest plot graph, CBT is the only method that provides a statistically significant difference in reducing depressive symptoms; the efficacy of other methods was not found to be statistically significant.

### Direct and indirect comparisons

Table 4 shows the results of direct and indirect comparisons between intervention methods. CBT stood out as the only intervention that statistically significantly reduced depressive symptoms compared to TAU. Other direct or indirect comparisons did not show statistically significant differences.

**Table 4:** Pairwise comparisons of all psychotherapeutic interventions

Comparison	Direct Comparison MD [95% CI]	Indirect Comparison MD [95% CI]
CBT vs. TAU	−3.219 [−5.906; −0.532]	—
CBT vs. MDMBI	—	−0.548 [−4.795, 3.699]
CBT vs. MT	—	−3.039 [−8.214, 2.135]
CBT vs. WBI	—	−1.189 [−6.231, 3.853]
CBT vs. WeChat-BHC	—	−1.108 [−7.235, 5.020]
MDMBI vs. TAU	−2.672 [−5.960, 0.617]	—
MDMBI vs. MT	—	−2.492 [−8.002, 3.019]
MDMBI vs. WBI	—	−0.642 [−6.028, 4.745]
MDMBI vs. WeChat-BHC	−0.560 [−4.977, 3.857]	—
MT vs. TAU	−0.180 [−4.602, 4.242]	—
MT vs. WBI	—	1.850 [−4.295, 7.995]
MT vs. WeChat-BHC	—	1.932 [−5.131, 8.994]
WBI vs. TAU	2.030 [−2.236, 6.296]	—
WBI vs. WeChat-BHC	—	0.082 [−6.885, 7.048]
WeChat-BHC vs. TAU	—	2.112 [−3.395, 7.619]

### Relative ranking of interventions (P-Score Analysis)

**Fig. 4:**
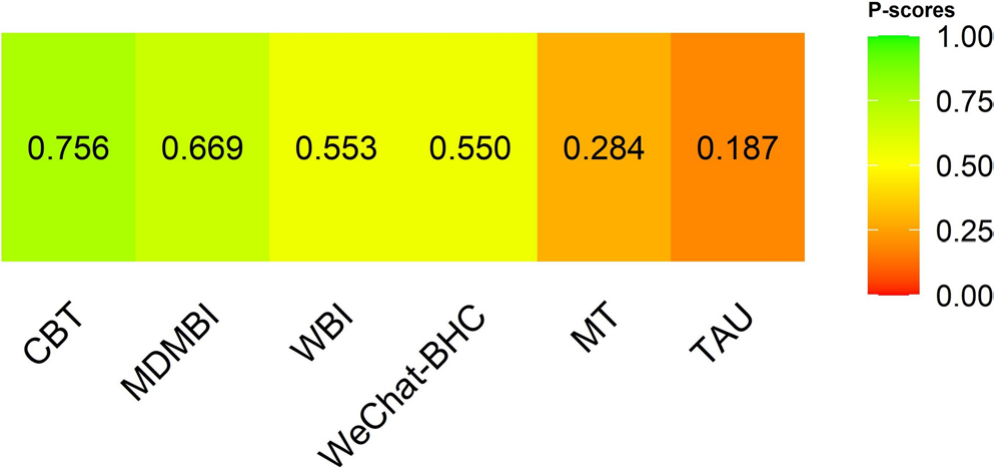
P-score

As a result of the NMA, the relative efficacy of psychotherapeutic interventions in reducing maternal depressive symptoms was ranked using P-score values (Fig. 4). These scores rate the relative efficacy of each intervention on a scale of 0 to 1. The P-score indicates the likelihood that an intervention is superior to other interventions, with values closer to 1 indicating greater efficacy. According to the P-score analysis, CBT (P-score = 0.756), which had the highest P-score value, was identified as the most efficacious among the psychotherapeutic approaches included in this study. The efficacy ranking of other intervention methods was as follows: from most to least efficacious: MDMBI (P-score = 0.669), WBI (P-score = 0.553), WeChat-BHC (P-score = 0.550), MT (P-score = 0.284), and TAU (P-score = 0.187).

However, this finding should be interpreted cautiously, as the confidence intervals for several interventions overlapped with those of TAU, suggesting that their relative inferiority cannot be firmly established.

### Publication bias and small study effects

**Fig. 5:**
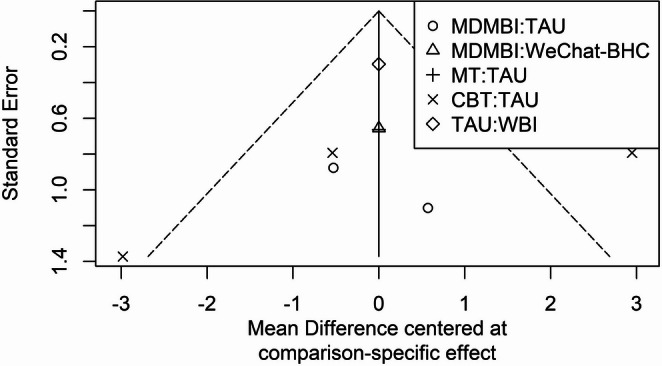
Funnel plot

Figure 5 shows the funnel plot graph created to evaluate possible publication bias and small study effects in the studies. In the graph, the horizontal axis represents the mean differences, while the vertical axis represents the standard error values. When examining the distribution of the studies, it was observed that the points were generally balanced around the central axis (mean effect size) and that there was no significant asymmetry. This supports the absence of significant publication bias in the NMA. However, the presence of studies with higher standard error values, particularly in the extreme regions (e.g., MT: TAU and CBT: TAU comparisons), indicates a significant level of heterogeneity (I² = 83.6%) in the analysis. In conclusion, the funnel plot graph revealed that there was no significant small study effect or systematic publication bias in the studies, but that heterogeneity among the studies should be taken into account.

## Discussion

This NMA study aims to provide a comprehensive perspective on current practice and evidence in this field by comparing the direct and indirect efficacy of different psychotherapeutic approaches to maternal depression. The NMA method used in the study is a statistical modeling approach that allows different treatment alternatives to be evaluated within a single analysis framework through both direct and indirect comparisons. According to the analysis results, only CBT provided a statistically significant reduction in depressive symptoms compared to TAU. This finding reflects CBT’s structured intervention strategies and its ability to produce concrete results in a short period of time (Beck 2011; Hofmann et al. 2012; Roman et al. 2020).

Although the studies included assessed the same construct (mother’s depressive symptoms) using the same instrument (EPDS), results were heterogeneous (I² = 83.6%), which likely reflects variability in both clinical and methodological aspects of the studies. Clinically, the studies varied in basic characteristics of participants (e.g., postpartum vs. antenatal population, and severity of depressive symptoms at baseline), intervention format (e.g., face-to-face, online, or mobile-based), and duration of therapy. Methodologically, there also existed variability in sample size, adherence, and definition of comparators (e.g., TAU vs. minimal care), which may have contributed to the statistical heterogeneity in the results. Either or both clinical and methodological variation is expected in studies of psychotherapy, and client variability should be characterized as an estimated reflection of real-world contexts of intervention implementation, rather than directly indicating variability in the direction of effects.

No strong evidence of publication bias was found in the funnel plot analysis. Although the present review included fewer than ten studies, the funnel plot was generated to provide a visual inspection of potential publication bias. However, it should be noted that funnel plot asymmetry tests have low power when fewer than ten studies are included, and the absence of asymmetry should therefore be interpreted cautiously (Sterne et al. 2011; Higgins et al. 2021). However, the presence of higher standard errors in comparisons with outlier values reinforces the severity of heterogeneity (Borenstein et al. 2021). Due to the nature of NMA, it is important to carefully evaluate both clinical and statistical heterogeneity levels and interpret the results in this context (Chaimani et al. 2013; Salanti 2012). Additionally, the P-score values used in the analysis provide a relative ranking of psychotherapeutic approaches rather than definitive evidence of superiority. CBT, which achieved the highest P-score (0.756), was identified as the most efficacious among the interventions included in this study, though this finding should be interpreted cautiously given the overlapping confidence intervals and high heterogeneity. Interventions such as MDMBI and WBI scored higher than TAU, highlighting their potential benefits. While these interventions did not show a statistically significant reduction in maternal depressive symptoms compared to TAU, this finding suggests that MT and WBI may still have potential therapeutic benefits. Therefore, P-score rankings should be viewed as indicative rather than conclusive, and future randomized controlled trials with more rigorous designs — including larger sample sizes, standardized intervention durations, and improved adherence monitoring — are needed to more precisely determine the efficacy of MDMBI and WBI interventions (Cristea et al. 2017).

The efficacy of CBT is based on its ability to restructure the automatic negative thoughts that underlie the mood disorders experienced by women, especially during the postpartum period. The role transition, sense of loss of control, and changes in self-image that women experience as they transition into motherhood make the thought patterns targeted by this type of therapy more visible and the intervention more effective (Cuijpers et al. 2023; Van Lieshout et al. 2020). Additionally, the individualized nature of CBT enables it to be effectively applied in different socio-cultural contexts. CBT interventions initiated during the antenatal period have been effective in reducing depressive symptoms that persist into the postpartum period and have facilitated the development of the individual’s emotional regulation skills. Furthermore, the applicability of CBT in digital environments has made it accessible to women who are unable to leave their homes or attend therapy in person due to childcare responsibilities. In this context, CBT can be positioned as a priority intervention method not only in the treatment of depression but also in its prevention (Andrews et al. 2018; Sockol 2015; Topkara and Özerdoğan 2022; Pettman et al. 2023).

Mindfulness-based therapies, another type of psychotherapeutic intervention, aim to develop awareness, acceptance, and the ability to connect with emotions, which make it easier to cope with stress. The goal of mindfulness therapies is to reduce mental load and break the cycle of depressive thoughts by enabling the individual to accept the present moment without judgment (Dimidjian and Segal 2015). In this study, although mindfulness practices showed a tendency toward a decrease in depressive symptoms, there was no statistically significant difference between them and TAU. The heterogeneity in application protocols, the duration of interventions, the training of practitioners, and the participants’ level of adherence to the application may be the reasons for this. Nevertheless, the literature emphasizes that such practices offer an alternative support option due to their low cost, lack of side effects, and ease of implementation in a group setting (Topkara and Özerdoğan 2022). Similarly, music therapy did not produce a significant difference in maternal depressive symptoms compared to TAU. The literature indicates that music therapy may exhibit variable effects depending on the dose-response relationship and that factors such as sufficient duration, content compatibility, and therapist expertise must be ensured for this therapy to be effective (Gold et al. 2009; Yang et al. 2019). It is thought that these factors may have contributed to the lack of a significant difference in maternal depressive symptoms between music therapy and TAU in this study. In this regard, it is important that future studies address the aforementioned factors in a detailed and systematic manner.

Web-based interventions (WBIs) are increasingly becoming the subject of research. They are particularly noteworthy as an important intervention alternative in rural areas or for individuals with limited social support (Andersson and Titov 2014). However, in this study, the effect of WBI on reducing maternal depressive symptoms was not statistically significant compared to TAU. Factors such as low user motivation, lack of personalization, and inadequate professional follow-up may have contributed to this outcome. This finding is important in highlighting the limitations of digital therapies (O’Mahen et al. 2014). While the WeChat-BHC health counseling program showed a trend toward reducing depression symptoms, it did not create a statistically significant difference compared to TAU. The fact that the WeChat-BHC application did not create a significant difference compared to TAU may be related to the application platform providing limited interaction between the client and the therapist and the inability to adapt interventions according to personal differences. However, in terms of high accessibility and low cost, such digital solutions can be used as supportive applications, especially in resource-constrained environments.

## Conclusion

This NMA study systematically evaluated the comparative efficacy of different psychotherapeutic interventions on maternal depression based on both direct and indirect data. NMA, a statistical analysis method, has the potential to produce strong evidence that can contribute to the decision-making process in clinical practice.

The results statistically support CBT as a safe, efficacious, and feasible method for combating maternal depression. Health professionals prioritizing evidence-based and structured CBT protocols in the delivery of health services will be an important step in protecting maternal and child mental health. Although some interventions other than CBT (MDMBI, WBI, and WeChat-BHC) attracted attention with their relative superiority scores in this study, these effects did not reach statistical significance.

Given the substantial heterogeneity observed across included studies, these findings should be interpreted with caution. Variability in study populations, intervention protocols, and outcome assessments may have influenced the pooled estimates and relative efficacy rankings. Therefore, it is recommended that randomized controlled studies with a stronger methodological basis, a large sample size, and cultural diversity be conducted to more clearly demonstrate the efficacy of these interventions.

## Data Availability

Data available on request from the corresponding author.
